# Phytoconstituents of traditional Himalayan Herbs as potential inhibitors of Human Papillomavirus (HPV-18) for cervical cancer treatment: An *In silico* Approach

**DOI:** 10.1371/journal.pone.0265420

**Published:** 2022-03-17

**Authors:** Deeksha Salaria, Rajan Rolta, Jyoti Mehta, Oladoja Awofisayo, Olatomide A. Fadare, Baljinder Kaur, Balvir Kumar, Renato Araujo da Costa, Shikha Rangra Chandel, Neha Kaushik, Eun Ha Choi, Nagendra Kumar Kaushik

**Affiliations:** 1 Faculty of Applied Sciences and Biotechnology, Shoolini University, Solan, Himachal Pradesh, India; 2 Department of Pharmaceutical and Medical Chemistry, University of Uyo, Uyo, Nigeria; 3 Organic Chemistry Research Lab, Department of Chemistry, Obafemi Awolowo University, Ile-Ife, Nigeria; 4 Department of Biotechnology, Punjabi University Patiala, Patiala, Punjab, India; 5 University Institute of Biotechnology, Chandigarh University, Mohali, Punjab, India; 6 Federal Institute of Education, Science, and Technology of Para, Para, Brazil; 7 Division of Microbiology, School of Pharmaceutical and Health Sciences, Career Point University, Hamirpur, Himachal Pradesh, India; 8 Department of Biotechnology, College of Engineering, University of Suwon, Hwaseong-si, South Korea; 9 Department of Electrical and Biological Physics, Plasma Bioscience Research Center & Applied Plasma Medicine Center, Kwangwoon University, Seoul, South Korea; Adama Science and Technology University, ETHIOPIA

## Abstract

Human papillomavirus (HPV) induced cervical cancer is becoming a major cause of mortality in women. The present research aimed to identify the natural inhibitors of HPV-18 E1 protein (1R9W) from Himalayan herbs with lesser toxicity and higher potency. In this study, one hundred nineteen phytoconstituents of twenty important traditional medicinal plants of Northwest Himalayas were selected for molecular docking with the target protein 1R9W of HPV-18 E1 Molecular docking was performed by AutoDock vina software. ADME/T screening of the bioactive phytoconstituents was done by SwissADME, admetSAR, and Protox II. A couple of best protein-ligand complexes were selected for 100 ns MD simulation. Molecular docking results revealed that among all the selected phytoconstituents only thirty-five phytoconstituents showed the binding affinity similar or more than the standard anti-cancer drugs viz. imiquimod (-6.1 kJ/mol) and podofilox (-6.9 kJ/mol). Among all the selected thirty-five phytoconstituents, eriodictyol-7-glucuronide, stigmasterol, clicoemodin and thalirugidine showed the best interactions with a docking score of -9.1, -8.7, -8.4, and -8.4 kJ/mol. Based on the ADME screening, only two phytoconstituents namely stigmasterol and clicoemodin selected as the best inhibitor of HPV protein. MD simulation study also revealed that stigmasterol and clicoemodin were stable inside the binding pocket of 1R9W, Stigmasterol and clicoemodin can be used as a potential investigational drug to cure HPV infections.

## 1. Introduction

Malignant tumors have cancerous cells that grow indefinitely and proliferate locally or to the nearby body parts. They can also metastasize to distant parts via the bloodstream or the lymphatic system and affects the liver, lungs, brain, bone, and other areas. Some of the viral infections including Epstein-Barr, Human immunodeficiency virus (HIV), Human papillomavirus (HPV), Human herpesvirus 8 (HHV-8), hepatitis B and C, etc. have been well documented in increasing the risks of various cancers. Cervical cancer is identified as the fourth most common malignancy among women worldwide and even ranked after breast cancer with 2·1 million cases, colorectal cancer with 0·8 million cases, and lung cancer with 0·7 million cases worldwide [1]. Each year 0.5 million women are diagnosed with cervical cancer [2].

Viral infections are responsible for enhancing the prevalence and as well as the risks associated with approximately 15–20% of the cancers in the total human population, whereby numerous viruses play a key role in the etiology of some malignant cancers at multiple stages. HPV infection is considered to be one of the common risk factors for the development of cervical cancer, wherein about 99% of the cases are associated with cervical cancer [3, 4]. HPV is a double-stranded DNA and a non-enveloped virus of the family Papovaviridae. It is 55 nm in diameter and has an icosahedral capsid comprising of 72 capsomeres, each one with at least two capsid proteins namely L1 and L2. HPV infection is widespread in humans and has been found in several animals as well, where it is unique to its host. It is reported that HPV is present in both symptomatic and asymptomatic populations and there are many more cancers that are cognate with HPV infections like anogenital, vulvar, vaginal, penile, head, and neck [5–8]. More than 120 HPV genotypes have been classified which infects human skin and among these only 13–15 types are associated with cancer involving high-risk and low-risk types depending on their oncogenic potential [9]. According to the earlier data, about 5% of the population in Europe is infected with HPV and 26% in the Sub-Saharan African countries [10], 21.4% in Eastern Europe, and 16.1% in Latin America [11]. In India, 6.3% of the population is infected with HPV [12], where 10.4% in Andhra Pradesh [13], 9.7% in Varanasi [6], 8.1% in Mumbai, 15.4% in New Delhi, 18.9% in Bangalore, 1.3% in Bhopal and 3.5% in Chennai [14] have been recorded. The high-risk group HPV genotypes are the primary cause for the development of pre-cancerous and cancerous cervix [15]. HPV-18 is one of the major cause of the development of cervical cancer in humans [11], whereas HPV-11 presents a low-risk for cervical cancer, usually causing benign hyperproliferative lesions as genital warts and oral papillomatosis. Transmission of the infection is caused by sexual contact which results in squamous intraepithelial lesions. Due to the immunological barriers, the induced lesions may disappear after 6 to 12 months, but in the case of chronic infections, it can lead to the development of cervical cancer [16].

The development of cervical cancer mainly depends upon the interference of the tumor suppressor proteins, p53 and pRB (retinoblastoma) with viral oncoproteins E6 and E7. A dysregulation in the cellular adhesion, cellular control, host cell immunomodulation, and genotoxicity is associated with the severity of the disease [17]. Infections can be cured by the use of chemotherapeutic agents which are non-specific to their targets or by the application of surgical and ablative techniques which are invasive and expensive alternatives [18]. Moreover, their availability is scarce to millions of patients, particularly in developing countries. Hence, one of the main alternatives to treat HPV-related diseases is the availability of highly effective natural therapeutics directed against the virus. Therefore, medicinal plants such as *Allium sativum*, *Artemisia annua*, *Berberis aristata*, *Bergenia ligulata*, *Cannabis sativa*, *Cymbopogon citrates*, *Dactylorhiza hatagirea*, *Moringa oleifera*, *Myristica fragrans*, *Oxalis corniculata*, *Picrorhiza kurroa*, *Piper nigrum*, *Pleurospermum brunonis*, *Podophyllum hexandrum*, *Rheum emodi*, *Thalictrum foliolosum*, *Thymus serpyllum*, *Trillium grandiflorum*, *Viola odorata*, and *Zanthoxylum armatum* which are traditionally used for the treatment of diabetes, gastrointestinal, hepatic, nervous, skin, and respiratory tract infections, and rheumatism [19–33], were investigated for their biochemical compositions. Earlier also, many natural and plant-based compounds have been identified as promising sources for developing cytotoxic agents for the treatment and prevention of cancer in recent years [34]. Stigmasterol, an active ingredient of *B*. *aristata* has also been shown to exert anti-angiogenic and anti-cancer effects. downregulation of TNF-alpha and VEGFR-2 [35]. Therefore, the present research is focused on to study the intraction of major phytocompounds of twenty important medicinal plants of North west Himalays with target protein of HPV-18 capsid protein L1 and to study the toxicity of active phytocomplounds docking.

## 2. Methodology

### 2.1 Bioinformatics tools

Computational facilities to perform in silico investigations of the important drug candidates included Open Babel GUI [36], UCSF Chimera 1.8.1 [37], PubChem (www.pubchem.com), RCSB PDB (http://www.rscb.org/pdb), Autodock 1.5.6/vina software [38] and discovery studio.

### 2.2 Ligand preparation

One hundred nineteen major phytoconstituents of twenty medicinal plants (*Allium sativum*, *Artemisia annua*, *Berberis aristata*, *Bergenia ligulata*, *Cannabis sativa*, *Cymbopogon citrates*, *Dactylorhiza hatagirea*, *Moringa oleifera*, *Myristica fragrans*, *Oxalis corniculata*, *Picrorhiza kurroa*, *Piper nigrum*, *Pleurospermum brunonis*, *Podophyllum hexandrum*, *Rheum emodi*, *Thalictrum foliolosum*, *Thymus serpyllum*, *Trillium grandiflorum*, *Viola odorata* and *Zanthoxylum armatum*) collected from the higher altitudes of Himachal Pradesh and Uttarakhand (India) were selected for the molecular docking studies. The 3-dimensional structures of the selected phytoconstituents and the two standard drugs of the HPV (podofilok and imiquimod) were downloaded from the PubChem database (www.pubchem.com) in.sdf formats. The.sdf files of the phytoconstituents were converted into PDB formats. All the selected ligands (phytoconstituents and drugs) were prepared using the open babel program from the command line on an Ubuntu terminal. The energy of all the selected phytocompounds was minimized by using chemdraw 3D 15.0. Phytoconstituents selected for the study, plant source, and their ethnomedicinal uses are enlisted in Table 1.

**Table 1:** Ethno-medicinal uses and plant sources of selected phytoconstituents.

Major Phytoconstituents Selected for anti-carcinogenicity	Source of Phytoconstituents	Ethno-medicinal uses
Alizarin, Aloe-emodin, Anthraquinone, Anthrarufin, Anthrone, Chryophanol, Clicoemodin, Dantron, Emodin, Gallic acid, Juglone, Physicon, Piceatannol, Quinizarin, Rubiadin	*Rheum emodi*	Rheumatism [39], wounds healing and fractures [19], treatment of boils [40].
3-Octanone, 8-Prenylnaringenin, Apigenin, Apigenin-7-O-glucoside, Bornyl acetate, Camphene, Camphor, Carvacrol, Caryophyllene oxide, Catechin, Eriodictyol, Eriodictyol-7-glucuronide, Geranyl acetate, Linalool, Myrcene, Rutin, Spathulenol, Taxifolin, Thymol, Trans-Nerolidol, α-Pinene, β-Bisabolene, β-Caryophyllene, β-Pinene, δ-Cadinene.	*Thymus serpyllum*	Cold, flu, indigestion, hair loss treatment, diabetes, and respiratory diseases [20]
Citral alpha, Citronellal, Geraniol, lemonene, Nerol, Terpinolene	*Cymbopogon citrates*	Inflammation, fever, nervous disorders, pyrea, wound healing, hypertension, bone fracture [21]
Glucomoringin, Niazinicin A, Niazinin, Pterygospermin	*Moringa oleifera*	Skin infections, anxiety, asthma, wounds, fever, diarrhea, and sore throats [22], its seeds are used for purifying water [23]
8-Oxyberberine, Berberine, Jatrorrhizine, Noroxyhydrastinine, Palmatine, Thalicarpine, Thalidasine, Thalirugidine, Thalirugine, Thalisopine, Thalrugosaminine, Thalrugosidine	*Thalictrum foliolosum*	Atonic dyspepsia, edema, skin diseases, and Jaundice [24].
Berbamine, Columbamine, Isocorydine, Lambertine, Lupeol, Oleanolic acid, Oxyberberine, Oxycanthine, Stigmasterol	*Berberis aristata*	Skin diseases, menorrhagia, diarrhoea, Jaundice, and piles [25]
Guaiol, Piperazine, Piperine, Piperolein A, Piperolein B, Sabinene, β-caryophyllene	*Piper nigrum*	Skin diseases, Cough and cold, snake bite, and poisoning [26, 27].
(E)-Ajoene, (Z)-Ajoene, Allicin, Alliin, Vinyllithium	*Allium sativum*	Skin diseases, Cough and cold [25]
4-terpinol, 9-epoxylignan, Camphene, Elemicin, Isoelemicin, Methoxyeugenol, Meso-dihydroguaiaretic acid, Myristicin, Sabinene, Safrole, Trimyristin	*Myristica fragrans*	Hypertension, antispasmodic, diarrhea, abdominal pain, fever, and vomiting [28].
Armatamide, Bergapten, Sesamin, Tambulin, Xanthyletin, β-Sitosterol-D-Glucoside	*Zanthoxylum armatum*	Dental problems, fever, dyspepsia, cold, cough, and abdominal pain [29].
3-O-b-galactopyranoside, Methyl a-L-arabinopyranoside, Sedanolide	*Pleurospermum brunonis*	Skin infection and fever [30].
Cannabigerol, Cannabinoids, Cannabinol, Tetrahydrocannabinol	*Cannabis sativa*	Intoxicant, analgesic, narcotic, stomachic, antispasmodic, anodyne, and sedative [31, 32].
Curcubitacine E, Pikuroside, Verbascoside	*Picrorhiza kurroa*	Improve appetite, and decoction of this with ajwain is used for the purification of blood and to cure skin infections [41].
Isovitexin, Oleic acid, Palmitic acid	*Oxalis corniculata*	Dyspepsia [30].
Artemisinin, Dihydroartemisinic acid	*Artemisia annua*	Fever, malaria cough, cold, and blood detoxifier [42].
Bergenin, Pyrogallol	*Bergenia ligulata*	Excessive uterine hemorrhage, diseases of the urinary bladder, dysentery, menorrhagia, splenic enlargement, and heart diseases [33].
Dactylorhin E	*Dactylorhiza hatagirea*	It is taken with milk in case of weakness [30].
Podophyllin	*Podophyllum hexandrum*	It is given to cattle for bloating prevention [30].
Octadecanoic acid	*Trillium grandiflorum*	Antiseptic, antispasmodic, diuretic poultice to the eye to reduce swelling, aching rheumatic joints [43, 44] & sore ear [45].
Emetine	*Viola odorata*	Respiratory & gastrointestinal illness, cough, cold, and fever [46].

### 2.3 Protein preparation

Human papillomavirus type (HPV-18 E1) protein (PDB ID: 1R9W) [47] was selected for molecular docking with major bioactive phytoconstituents from the selected Himalayan medicinal plants as listed in Table 1 to identify its potential inhibitor. Podofilok and Imiquimod is used as drug target because of its small surface area and functional significance, the E1 dimerization interface is an obvious target for a small molecule disrupting DNA replication [47]. The 3-dimensional monomeric structure of HPV-18 E1 protein (1R9W) was downloaded from the protein databank (http://www.rscb.org/pdb). It consists of a pentamer structure, and its chain A was extracted for docking using Pymol software. Protein was prepared by using autoDock 1.5.6 program, during protein preparation all the water molecules were deleted, polar hydrogens were and kollaman charge (5.0) is added. The active site was predicted manually by grid box analysis (grid dimensions x = 40, y = 40, z = 40 Å, centered at x, y, z = 21.25, -0.773, 12.365 Å), TYR121, ASP124, VAL 126, SER129, TYR132, GLY133, GLY134, ASN135, PRO136, ASN259, ALA284, SER285, SER286, TYR288 are the key amino acids present in the active site of the protein.

### 2.4 Molecular docking using herbal phytoconstituents

The molecular docking of the selected ligands with the catalytic triad of the 1R9W protein was performed using AutoDock 1.5.6 program and responses were saved as a PDBQT file. Molecular docking was performed using vina Perl script [38] and the best conformation with the lowest docking energy was chosen. The.pdb complexes of the protein (1R9W) and ligands were analyzed on a Discovery Studio (https://discover.3ds.com/d) to study the list of interactions between the selected protein and ligands.

### 2.5 *In silico* pharmacokinetics and toxicity prediction of bioactive active phytoconstituents

ADME/T screening of the 35 selected bioactive phytoconstituents was done by using online web servers such as SwissADME (Molecular Modeling Group of the Swiss Institute of Bioinformatics, Lausanne, Switzerland), admetSAR (Laboratory of Molecular Modeling and Design, Shanghai, China), and ProTox (Charite University of Medicine, Institute for Physiology, Structural Bioinformatics Group, Berlin, Germany) [48–53].

### 2.6 MD simulation of protein-ligand complexes

To gain deeper insights into the binding interactions of 1R9W protein with clicoemodin and stigmasterol, MD simulations were performed that accelerated the graphics processing unit (GPU) in Amber18 [54]. Amber generalized force field (GAFF) was assigned to the ligands, whilst amber ff14SB to the protein [55, 56]. The atomic charges of the ligands were calculated using the restrained electrostatic potential (RESP) protocol at the HF/6-31G* level of theory 31, 32 using Gaussian 09 software [57]. The protonation states of the ionizable residues of protein structures were analyzed by pKa calculation at neutral pH using the H++ server. All the systems were prepared using tLeap program. Each system was neutralized with sodium ions and solvated using a cubic water box with TIP3P model. To eliminate the stearic clashes, each system was subjected to a total of four minimizations. First, all the sodium ions and water molecules were minimized by 2000 steps of steepest descent, followed by 3000 steps of the conjugate gradient algorithm. Consecutively, all the hydrogen and water molecules were relaxed using the same protocol. Finally, the whole system was energy-minimized for 5000 steps of steepest descent plus 5000 steps of conjugate gradients. The heat of the system was started from 0 to 300 K while running 200 ps of MD and, next, 300 ps of the density equilibration with position restraints on the protein atoms at a constant volume. Before performing the production step, all the protein-ligand systems were equilibrated with10 ns of MD without any positional restraints at a constant pressure. The temperature was maintained at 300 K by coupling to a Langevin thermostat using a collision frequency of 2 cm^-1^. A cutoff of 10 Å was employed for unbonded interactions, the Particle Mesh Ewald (PME) [56] method and the SHAKE [57] algorithm were used to restrict the bond lengths involving hydrogen atoms. Finally, the MD simulations (productions) were performed using 100 ns at a temperature of 300 K. The generated trajectories were used to analyze the behavior of each complex and to access the stability of the system. The deviations of the protein and protein-ligand complex system were analyzed by calculating the root mean square deviation (RMSD), root mean square fluctuation (RMSF), a radius of gyration (RG), and solvent accessible surface area (SASA) parameters [58, 59].

Furthermore, the total free energy of binding, the free energy of solvation (polar þ non-polar solvation energies), and the potential energy (electrostatic and van der Waal’s interactions) of each protein-ligand complex were calculated by molecular mechanics using Poisson–Boltzmann Surface Area (MM-PBSA) method [60]. The last 10 ns of the MD trajectory were taken for the calculation of MM-PBSA.

## 3. Results

### 3.1 Molecular docking of some medicinally important phytoconstituents with target protein of HPV-18 (1R9W)

One hundred nineteen major phytoconstituents of twenty important medicinal plants of Northwest Himalayas are selected based on their reported medicinal attributes as evidenced in the earlier literature. These are widely recommended in the Ayurvedic/Unani medicines for the treatment of diabetes, gastrointestinal, hepatic, nervous, skin, and respiratory tract infections, and rheumatism (as summarized in Table 1). 3D structures of these phytoconstituents and their target proteins were retrieved from PubChem and PDB databases. Molecular docking results showed that out of one hundred nineteen phytoconstituents, only thirty-five phytoconstituents of twelve medicinal plants showed docking scores more than the standard drugs imiquimod (-6.1 kJ/mol) and podofilok (-6.9 kJ/mol) interactions with HPV-18 (1R9W). Important phytoconstituents with their respective docking score, plant sources, and interactive amino acids are summarized in Table 2 and S1 Table. From this study, we found that six phytoconstituents of *Thalictrum foliolosum* and *Thymus serpyllum*, five of *Berberis aristata*, four of *Rheum emodi*, three each from *Cannabis sativa* and *Picrorhiza kurroa*, two each from *Moringa oleifera* and *Zanthoxylum armatum*, and one each from *Myristica fragrans*, Oxalis corniculata, *Piper nigrum*, and Pleurospermum brunonis showed docking score more than the standard anti-cancerous drugs.

**Table 2:** The table exhibits important bioactive phytoconstituents of herbal plants their docking with HPV-18 (1R9W) and interactive amino acids.

S.no	Phytoconstituents/Standard drug	Plant source	Docking score (-kJ/mol)	Interactive amino acids	
				H- bonds	Hydrophobic interactions
1.	Chryophanol	*Rheum emodi*	-7	-	ALA 213, MET 214, LYS 210, TRP 278, PHE 248, CYS 311, GLY 249
2.	Emodin		-7.1	LYS 20	GLY 249, PHE 248, TRP 278, CYS 311, ALA 213, MET 214
3.	Rubiadin		-6.9	-	GLY 249, CYS 311, TRP 278, LYS 210, PHE 248, ALA 213, MET 214
4.	Clicoemodin		-8.4	ASN 335, SER 337	GLY 341, ILE 333, SER 334, GLY 332, TYR 329, GLYU 338, VAL 339, THR 343, GLN 348
5.	Apigenin	*Thymus serpyllum*	-7.1	TYR 268	HIS 270, VAL 339, GLY 332, THR 343, GLN 348, TYR 329, ILE 347, TRP 328
6.	Catechin		-7.5	TYR 268, TRP 328	ILE 333, GLN 348, TYR 329, ILE 347, GLY 332, THR 343, HIS 270
7.	Eriodictyol-7-glucuronide		-9.1	VAL 339, LYS 259, THR 343	ILE 333, TYR 329, HIS 270, TYR 268, GLY 332, SER 337, GLU 338
8.	Eriodictyol		-7.5	TYR 268	TYR 329, ILE 347, TRP 328, GLY 332, THR 343, HIS 270, MET 340
9.	Rutin		-8.3	TYR 268, TRP 328, THR 343, GLY 341, SER 337, ASN 335	HIS 270, ASP 342, GLY 332, MET 340, ILE 347, TYR 329, ILE 333, SER 334, GLU 338
10.	Taxifolin		-7.2	TYR 268, TRP 328	GLN 348, THR 343, TYR 329, ILE 347, GLY 332, HIS 270, MET 340
11.	Glucomoringin	*Moringa oleifera*	-7.4	THR 343, GLY 332, TYR 268	TYR 329, VAL 339, ILE 333, PRO 344, HIS 270, TRP 328, ILE 347, ARG 349, GLY 341, ASP 342, GLN 348, THR 351, ILE 353
12.	Pterygospermin		-7.6	-	VAL 323, LEU 224, TYR 327, GLY 223, LEU 326, THR 331, ARG 330
13.	8-Oxyberberine	*Thalictrum foliolosum*	-7.4	-	LYA 210, ALA 213, MET 214, TRP 278, PHE 248, GLY 249, VAL 307, CYS 311, THR 310
14.	Thalicarpine		-7.9	-	TRP 328, ILE 333, VAL 339, ILE 347, TYR 329, THR 343, GLN 348, GLY 332, TYR 268, HIS 270, MET 340, GLY 341
15.	Thalidasine		-7.8	ARG 287	LEU 319, LEU 350, TYR 346, ARG 349, ARG 320, CYS 240, THR 241, ASP 242, ARG 231, LEU 267
16.	Thalirugidine		-8.4	-	HIS 270, ILE 347, TYR 329, TYR 268, GLN 348, THR 343, GLY 341, ASP 342, ILE 333, VAL 339, GLY 332, SER 337, GLU 338
17.	Thalirugine		-8	ASP 125, TYR 288	ASP 124, TYR 123, ALA 284, SER 129, VAL 126, GLU 127, PHE 257, HIS 290, VAL 289
18.	Thalrugosaminine		-7.8	-	VAL 280, CYS 273, PRO 252, GLU 256, ALA 255, VAL 280, LEU 274, ASP 275, ILE 336, ASN 335
19.	Berbamine	*Berberis aristata*	-8.3	THR 212	GLY 180, LEU 210, GLY 265, PRO 183, MET 205, VAL 289, ASP 216, ARG 260, ALA 261, ILE 287, ASN 121, GLY 262, ASN 213
20.	Berberine		-6.9	THR 241, ARG 287	CYS 240, TRP 346, GLU 345, ARG 349, LEU 319, ASP 242, ARG 320, LEU 350
21.	Lupeol		-8.2	ASP 228	TYR 222, THR 331, TYR 327, ARG 330, LEU 224, VAL 323
22.	Oleanolic acid		-8	VAL 323	LEU 326, ARG 330, THR 331, GLN 272, LEU 274, ILE 336, EU 224, TYR 323, TYR 222
23.	Stigmasterol		-8.7	-	HIS 270, TYR 268, ILE 333, VAL 339, GLY 332, TYR 329, GLN 348
24.	Piperolein B	*Piper nigrum*	-6.9	-	SER 334, THR 331, GLY 223, TYR 222, ARG 330, LEU 326, TYR 327, VAL 323, LEU 224
25.	9-epoxylignan	*Myristica fragrans*	-7	GLY 341, THR 343	MET 340, VAL 339, ILE 347, TYR 329, ILE 333, GLY 332, GLN 348
26.	Armatamide	*Zanthoxylum armatum*	-7.2	-	LYS 210, MET 214, PRO 308, ALA 213, TRP 278, PHE 248, CYS 311, GLY 249, VAL 307, VAL 250, LEU 305, ASN 251, HIS 306
27.	β-Sitosterol-D-Glucoside		-7.7	-	ASP 24, ARG 29, PHE 33, GLN 311, LYS 312, GLN 311, GLN 373, PHE 464, GLN 467, SER 468
28.	3-O-b-galactopyranoside	*Pleurospermum brunonis*	-7	GLY 341, THR 343	GLN 348, ILE 333, VAL 339, GLY 332, GLN 348, ILE 347, TYR 268, HIS 270, TRP 328, PRO 344
29.	Cannabigerol	*Cannabis sativa*	-7.2	-	TYR 222, GLN 272, TYR 327, LEU 326, ARG 330, VAL 323, LEU 224, ILE 336, LEU 274, THR 331
30.	Cannabinol		-7.1	GLY 223	LEU 326, THR 331, ARG 330, TYR 222, LEU 274, GLN 272, VAL 323, TYR 327, LEU 224
31.	Tetrahydrocannabinol		-7.3	-	ASP 228, GLY 223, TYR 222, VAL 323, LEU 224, TYR 327, THR 331, GLN 272, LEU 274, LEU 326, ARG 330, ILE 336
32.	Curcubitacine E	*Picrorhiza kurroa*	-7.7	ASP 220, SER 225	GLY 223, LYS 219, THR 227, ALA 216, LEU 215, PHE 226
33.	Pikuroside		-7.7	THR 343, SER 334, ASN 335, SER 337, LYS 259, VAL 339	ILE 347, TYR 268, HIS 270, GLY 332, TYR 329, MET 340, GLU 338, GLN 263, THR 260
34.	Verbascoside		-7.8	THR 241, TRP 346, GLU 345, ARG 287, ASP 342	CYS 240, ASP 242, ARG 231, PRO 344, TYR 268, GLY 341
35.	Isovitexin	*Oxalis corniculata*	-7.9	VAL 339, ASN 335, ILE 333, GLY 332	GLU 338, SER 337, SER 334, TYR 268, THR 343, GLN 348
36.	Podofilok	Standard drugs	-6.9	-	GLY 341, MET 340, VAL 339, HIS 270, TRP 328, TYR 329, GLY 332, THR 343, ILE 333
37.	Imiquimod		-6.1	-	GLN 348, TYR 329, ILE 347, TRP 328, HIS 270, TYR 268, THR 343, VAL 339, GLY 332

**Fig 1:**
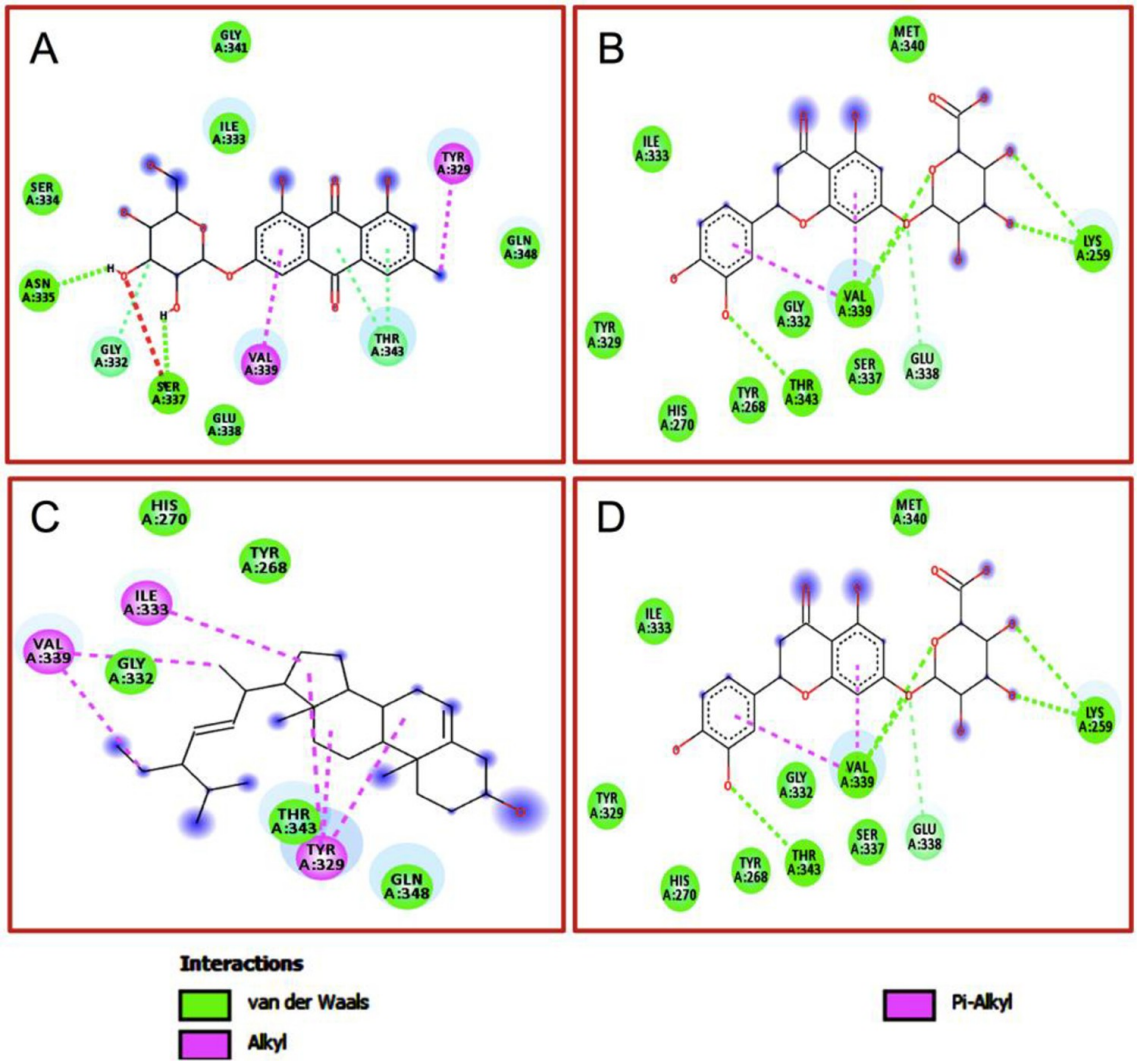
2D structures of active phytoconstituents in complexation with 1R9W: where, (A) Thalirugonidine, (B) Clicoemodin, (C) Eriodictyol-7, and (D) Stigmasterol.

**Fig 2:**
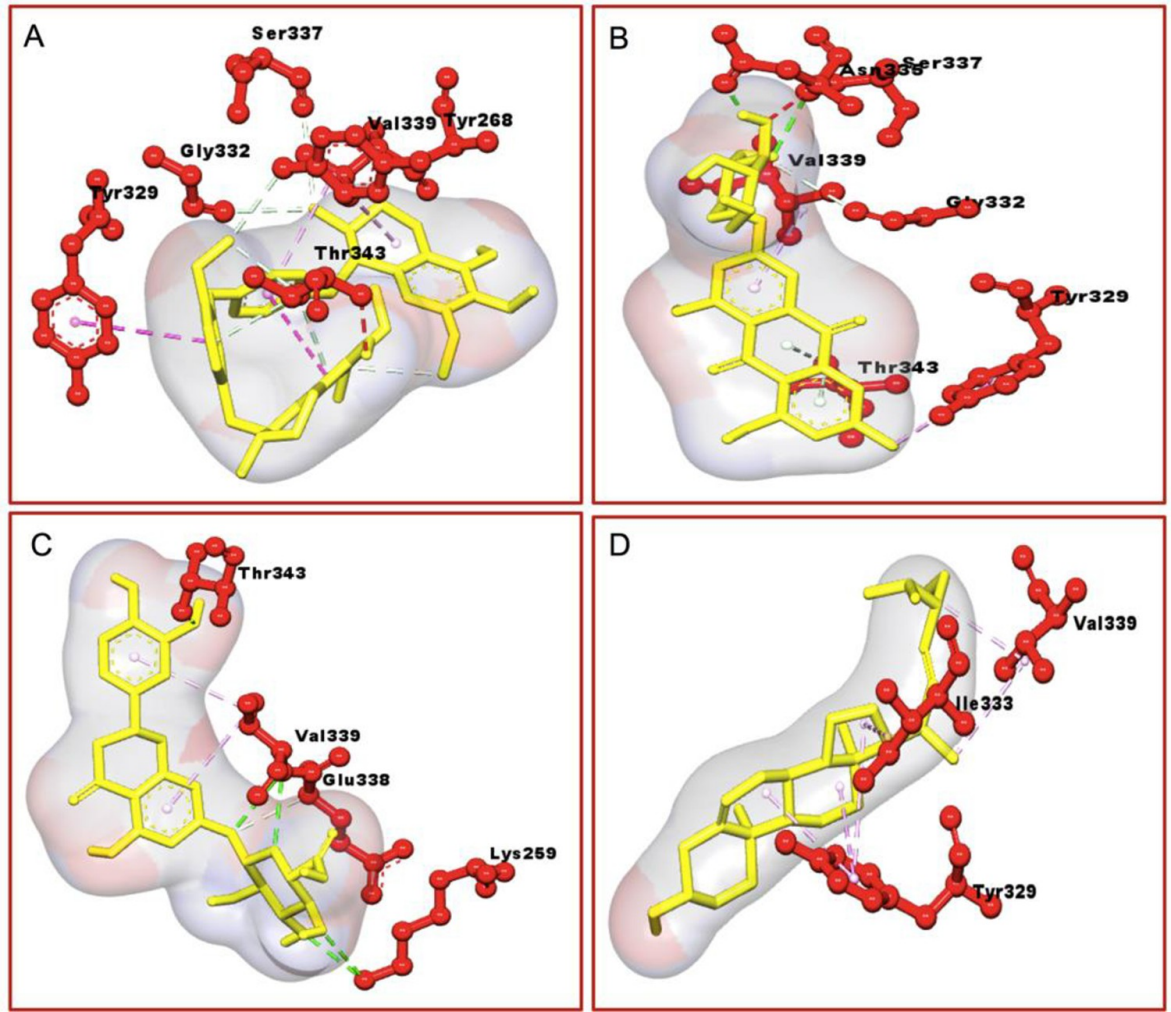
3D structure of active phytoconstituents in complex with 1R9W: (A) Thalirugonidine, (B) Clicoemodin, (C) Eriodictyol-7, and (D) Stigmasterol.

Among all the active thirty-five phytoconstituents eriodictyol-7-glucuronide of *Thymus serpyllum* showed the best interactions with a docking score of -9.1 kJ/mol followed by stigmasterol (-8.7 kJ/mol) of *Berberis aristata*, clicoemodin (-8.4 kJ/mol) of *Rheum emodi*, and thalirugidine (-8.4 kJ/mol) of *Thalictrum foliolosum*. Eriodictyol-7-glucuronide makes hydrogen bonds with VAL 339, LYS 259, THR 343 and showed hydrophobic interactions with ILE 333, TYR 329, HIS 270, TYR 268, GLY 332, SER 337, GLU 338, while stigmasterol showed hydrophobic interactions with HIS 270, TYR 268, ILE 333, VAL 339, GLY 332, TYR 329, and GLN 348, clicoemodin makes hydrogen bonds with ASN 335, SER 337 and showed hydrophobic interactions with GLY 341, ILE 333, SER 334, GLY 332, TYR 329, GLYU 338, VAL 339, THR 343, GLN 348 while thalirugidine didn’t make any hydrogen bond, it only showed hydrophobic interactions with HIS 270, ILE 347, TYR 329, TYR 268, GLN 348, THR 343, GLY 341, ASP 342, ILE 333, VAL 339, GLY 332, SER 337, GLU 338 (Table 2). 2D and 3D structures of the best complexes are represented in Figs 1 and 2. The remaining phytoconstituents with higher docking scores such as bornyl acetate, cannabinoids, caryophyllene oxide, dactylorhin E1, dactylorhin E2, dactylorhin E3, dactylorhin E4, dihydroatemisinic acid, gallic acid, geranylacetate 2, geranylacetate, meso-dihydrogualaretic acid, methy a-L-arabinopyranoside, niazinicin A, oleanolic acid, oleic acid, palmitic acid, piperolin A, piperolin B and podophyllin do not show any interaction with HPV 18 (1R9W).

### 3.2 *In silico* pharmacokinetics and toxicity prediction of the bioactive phytoconstituents

The pharmacokinetics and toxicity of pharmacologically important phytoconstituents were analyzed *in silico* using computational facilities of SWISS ADME, admetSAR, and Protox II servers. Thirty-five active phytoconstituents of twelve medicinal plants and two standard drugs (Podofilox and Imiquimod) were selected for the pharmacokinetics and toxicity studies. Among all the thirty-five phytoconstituents, only 29 phytoconstituents follow Lipinski’s rule of five, and phytoconstituents such as 3-O-b-galactopyranoside, eriodictyol-7-glucuronide, glucomoringin, pikuroside, rutin, and verbascoside do not follow Lipinski’s rule of five, therefore these cannot be used as investigational drug candidates. Whereas 30 phytocompounds follows the Veber’s rule. Phytocompounds such as 3-O-b-galactopyranoside, 8-Oxyberberine, Apigenin, Armatamide, Catechin, Clicoemodin, Clicoemodin, Eriodictyol-7-glucuronide, Eriodictyol, Glucomoringin, Isovitexin, Pikuroside, Piperolein B, Soluble, Rutin, Taxifolin, Verbascoside are water soluble. The GI-absorption of 8-Oxyberberine, 9-epoxylignan, Apigenin, Armatamide, Berbamine, Berberine, Cannabigerol, Cannabinol, Catechin, Chrysophanol, Clicoemodin, Emodin, Eriodictyol are found high. All the active phytocompounds were found non-carcinogenic in nature. Chrysophanol and Oleanolic acid are found hepetotoxic in nature. Eriodictyol, Berberine, Curcubitacine E, Armatamide, 9-epoxylignan, 8-Oxyberberine, 3-O-b-galactopyranoside Phytocompounds showed Carcinogenicity.Protox II server predict that phytocompounds such as 3-O-b-galactopyranoside, 8-Oxyberberine, 9-epoxylignan, Armatamide, Berbamine, Berberine, Cannabinol, Chrysophanol, Clicoemodin, Curcubitacine E, Eriodictyol-7-glucuronide¸ Glucomoringin, Lupeol, Lupeol, Pikuroside, Piperolein B, Rubiadin, Rutin, Stigmasterol, Tetrahydrocannabinol, Thalicarpine, Thalidasine, Thalirugidine, Thalirugine, Thalisopine, Thalrugosaminine, Thalrugosidine, Verbascoside and β-Sitosterol Glucoside are found immunotoxic in nature. 8-Oxyberberine, Armatamide, Berbamine, Berberine, Clicoemodin, Emodin, Isovitexin, Rubiadin, Taxifolin, Thalicarpine, Thalidasine, Thalirugidine, Thalirugine, Thalisopine, Thalrugosaminine, Thalrugosidine are found mutagenic in nature. 3-O-b-galactopyranoside, 8-Oxyberberine, Berberine are cytotoxic in nature. Results are summarized in Table 3 and S1–S3 Figs.

**Table 3:** Pharmacokinetics and toxicity prediction of the active phytoconstituents of Himalayan herbs.

Phyto-compounds	SWISS ADME							Admet SAR						Protox-II					
	1	2	3	4	5	6	7	8	9	10	11	12	13	14	15	16	17	18	19
3-O-b-galactopyranoside	-2.09	Soluble	Low	No	No	182.4	No	-	+	-	Non-carcinogen	-2.93	2.5194 (III)	500 (class 5)	Inactive	Inactive	Active	Inactive	Active
8-Oxyberberine	3.05	Soluble	High	Yes	Yes	58.92	No	+	+	+	Non-carcinogen	-2.79	2.40(III)	400 (class 4)	Inactive	Active	Active	Active	Active
9-epoxylignan	2.99	Moderately soluble	High	Yes	Yes	77.38	No	+	+	+	Non-carcinogen	-2.73	2.48 (III)	2000 (class 4)	Inactive	Inactive	Active	Inactive	Inactive
Apigenin	2.11	Soluble	High	Yes	Yes	90.9	Yes	+	-	-	Non-carcinogen	-2.77	1.14(III)	2500 (class 5)	Inactive	Inactive	Inactive	Inactive	Inactive
Armatamide	3.26	Soluble	High	Yes	Yes	56.79	No	+	+	+	Non-carcinogen	-3.30	1.74 (III)	1000 (class4)	Inactive	Active	Active	Active	Inactive
Berbamine	5.16	Poorly soluble	High	Yes	Yes	72.86	No	+	+	+	Non-carcinogen	-2.63	2.82 (III)	1700 (class 4)	Inactive	Inactive	Active	Active	Inactive
Berberine	2.53	Moderately soluble	High	Yes	Yes	40.8	No	+	+	+	Non-carcinogen	-2.97	1.54(III)	200 (class3)	Inactive	Active	Active	Active	Active
Cannabigerol	5.74	Poorly soluble	High	Yes	Yes	40.46	No	+	+	+	Non-carcinogen	-3.42	2.52(III)	500 (class4)	Inactive	Inactive	Inactive	Inactive	Inactive
Cannabinol	5.21	Moderately soluble	High	Yes	Yes	29.46	No	+	+	+	Non-carcinogen	-3.97	2.99 (III)	13500 (class6)	Inactive	Inactive	Active	Inactive	Inactive
Catechin	0.85	Soluble	High	Yes	Yes	110.3	Yes	+	-	-	Non-carcinogen	-3.10	2.14 (IV)	10000 (class6)	Inactive	Inactive	Inactive	Inactive	Inactive
Chrysophanol	2.38	Moderately soluble	High	Yes	Yes	74.6	No	-	-	+	Non-carcinogen	-3.12	2.91(II)	1190 (class4)	Active	Inactive	Active	In active	In active
Clicoemodin	0.19	Soluble	High	Yes	No	178.9	No	+	-	-	Non-carcinogen	-2.36	2.82(III)	3000 (class5)	Inactive	Inactive	Active	Active	Inactive
CurcubitacineE	3.47	Moderately soluble	Low	Yes	Yes	138.2	No	+	+	-	Non-carcinogen	-4.50	4.81 (I)	340 (class4)	Inactive	Active	Active	Inactive	Inactive
Emodin	1.87	Soluble	High	Yes	Yes	94.83	Yes	+	-	+	Non-carcinogen	-3.01	2.56(III)	5000 (class5)	Inactive	In active	In active	Active	Inactive
Eriodictyol-7-glucuronide	-0.37	Soluble	Low	No	No	203.4	No	+	-	-	Non-carcinogen	-3.71	1.85 (II)	2300 (class5)	Inactive	Inactive	Active	Inactive	Inactive
Eriodictyol	1.45	Soluble	High	Yes	Yes	107.2	Yes	+	-	-	Non-carcinogen	-3.44	2.41 (II)	2000 (class4)	Inactive	Active	Inactive	Inactive	Inactive
Glucomoringin	-1.82	Very soluble	Low	No	No	278.9	No	-	+	-	Non-carcinogen	-3.16	2.56(III)	3750 (class5)	Inactive	Inactive	Active	Inactive	Inactive
Isovitexin	0.05	Soluble	Low	Yes	No	181.0	No	+	-	-	Non-carcinogen	-2.39	2.81(IV)	159 (class3)	Inactive	Inactive	Inactive	Active	Inactive
Lupeol	7.31	Poorly soluble	Low	Yes	Yes	20.23	No	+	+	-	Non-carcinogen	-4.41	3.85 (III)	2000 (class4)	Inactive	Inactive	Active	Inactive	Inactive
Oleanolic acid	6.06	Poorly soluble	Low	Yes	Yes	57.53	No	+	-	+	Non-carcinogen	-4.38	2.70(III)	2000 (class4)	Active	Active	Active	Inactive	Inactive
Pikuroside	-1.53	Very soluble	Low	No	No	214.0	No	+	-	-	Non-carcinogen	-1.98	2.87(III)	2260 (class5)	Inactive	Inactive	Active	Inactive	Inactive
Piperolein B	4.47	Soluble	High	Yes	Yes	38.77	No	+	+	+	Non-carcinogen	-3.51	1.68(III)	1000 (class4)	Inactive	Active	Active	Inactive	Inactive
Pterygospermin	3.75	Moderately soluble	High	Yes	Yes	89.12	No	+	+	-	Non-carcinogen	-4.32	2.41(III)	898 (class4)	Inactive	Inactive	Inactive	Inactive	Inactive
Rubiadin	2.23	Soluble	High	Yes	Yes	74.6	Yes	+	-	+	Non-carcinogen	-3.11	2.09(III)	7000 (class5)	Inactive	Inactive	Active	Active	Inactive
Rutin	-1.12	Soluble	Low	No	No	269.4	No	+	-	-	Non-carcinogen	-2.77	2.59(III)	5000 (class5)	Inactive	Inactive	Active	Inactive	Inactive
Stigmasterol	6.96	Poorly soluble	Low	Yes	Yes	20.23	No	+	+	+	Non-carcinogen	-4.70	3.28 (I)	890 (class4)	Inactive	Inactive	Active	Inactive	Inactive
Taxifolin	0.63	Soluble	High	Yes	Yes	127.4	Yes	+	-	-	Non-carcinogen	-2.999	3.26(II)	2000 (class4)	Inactive	Active	Inactive	Active	Inactive
Tetrahydrocannabinol	5.28	Poorly soluble	High	Yes	Yes	29.46	No	+	+	+	Non-carcinogen	-4.321	3.61(III)	482 (class4)	Inactive	Inactive	Active	Inactive	Inactive
Thalicarpine	5.68	Poorly soluble	High	Yes	Yes	80.32	No	+	+	-	Non-carcinogen	-3.26	2.37(III)	1500 (class4)	Inactive	Inactive	Active	Active	Inactive
Thalidasine	5.34	Poorly soluble	High	Yes	No	71.09	No	+	+	-	Non-carcinogen	-2.81	2.41(III)	1700 (class4)	Inactive	Active	Active	Active	Inactive
Thalirugidine	5.2	Poorly soluble	High	Yes	Yes	102.3	No	+	+	-	Non-carcinogen	-2.83	2.38(III)	1180 (class4)	Inactive	Inactive	Active	Active	Inactive
Thalirugine	5.26	Poorly soluble	High	Yes	No	93.09	No	+	+	-	Non-carcinogen	-2.83	2.25(III)	1180 (class4)	Inactive	Inactive	Active	Active	Inactive
Thalisopine	5.09	Poorly soluble	High	Yes	Yes	82.09	No	+	+	+	Non-carcinogen	-2.50	3.09 (III)	1700 (class4)	Inactive	Inactive	Active	Active	Inactive
Thalrugosaminine	5.43	Poorly soluble	High	Yes	Yes	71.09	No	+	+	+	Non-carcinogen	-2.86	2.53 (III)	1700 (class4)	Inactive	Inactive	Active	Active	Inactive
Thalrugosidine	5.05	Poorly soluble	High	Yes	Yes	82.09	No	+	+	+	Non-carcinogen	-2.63	2.55(III)	1700 (class4)	Inactive	Inactive	Active	Active	Inactive
Verbascoside	-0.43	Soluble	Low	No	Yes	245.29	No	+	-	-	Non-carcinogen	-1.67	2.79(III)	5000 (class5)	Inactive	Inactive	Active	Inactive	Inactive
β-Sitosterol Glucoside	5.51	Moderately soluble	Low	Yes	Yes	99.8	No	+	+	-	Non-carcinogen	-4.40	2.91 (III)	1190 (class 4)	Active	Inactive	Active	In active	In active
Podofilox	2.27	Soluble	High	Yes	Yes	92.98	No	+	+	+	Non-carcinogen	-3.12	3.00(III)	100 (class 3)	Inactive	Active	Active	Inactive	Inactive
Imiquimod	2.34	Soluble	High	Yes	Yes	56.73	No	+	+	+	Non-carcinogen	-4.03	2.09 (III)	300 (class3)	Inactive	Active	Inactive	Active	Inactive

where, 1-Consensus log P o/w, 2-water solubility, 3-GI-absorption, 4-Lipinski rule, 5- Veber’s rule, 6-TPSA (Å2), 7-Lead likeness, 8-Human intestinal absorption, 9-Blood brain barrier penetration, 10-Caco-2 cell permeability, 11-Carcinogens, 12-Aquatic & terrestrial toxicity (Log S value), 13-Rate acute toxicity (LD50), 14-LD50,(mg/kg), 15-Organ toxicity hepatotoxicity, 16-Carcinogenicity, 17-Immunotoxicity, 18-Mutagenicity, 19-Cytotoxicity, “+” Positive and “-” Negative.

Based on the molecular docking drug-likeness data and toxicity data, two best phytoconstituents (stigmasterol from *Berberis aristata* and clicoemodin from *Rheum emodi*) were selected for further MD simulation analysis.

### 3.3 MD simulation of protein-ligand complexes

**Fig 3:**
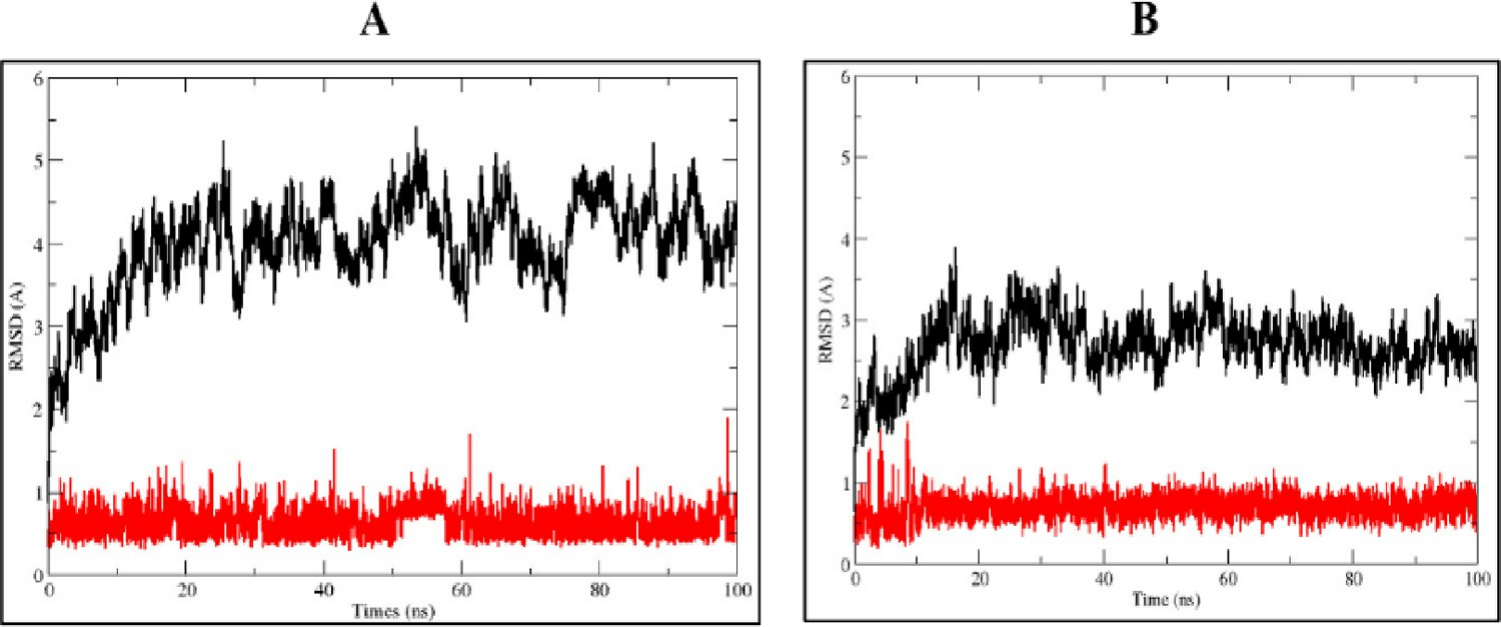
RMSD of active phytoconstituents in complexation with 1R9W: A- Stigmasterol and B-Clicoemodin.

The structural stability of the protein-ligand complex was determined using RMSD analysis during a specific time (100 ns). Molecular dynamic simulation of the selected protein-ligand complexes revealed that stigmasterol is stable inside the binding pocket of 1R9W. RMSD of stigmasterol was stable from the beginning of the simulation (0 ns) and remain stable up to 100ns period, which indicates fitness of the ligand inside the binding pocket of the target protein. RMSD has a little fluctuation between 0.5–1 Å, which is acceptable (Fig 3A). Similarly, the RMSD of clicoemodin, in complex with 1R9W is stable from the beginning (0 ns) and remains stable up to 100 ns periods, and only a little fluctuation was observed which is in the acceptable range (0.5–1 Å) (Fig 3B).

**Fig 4:**
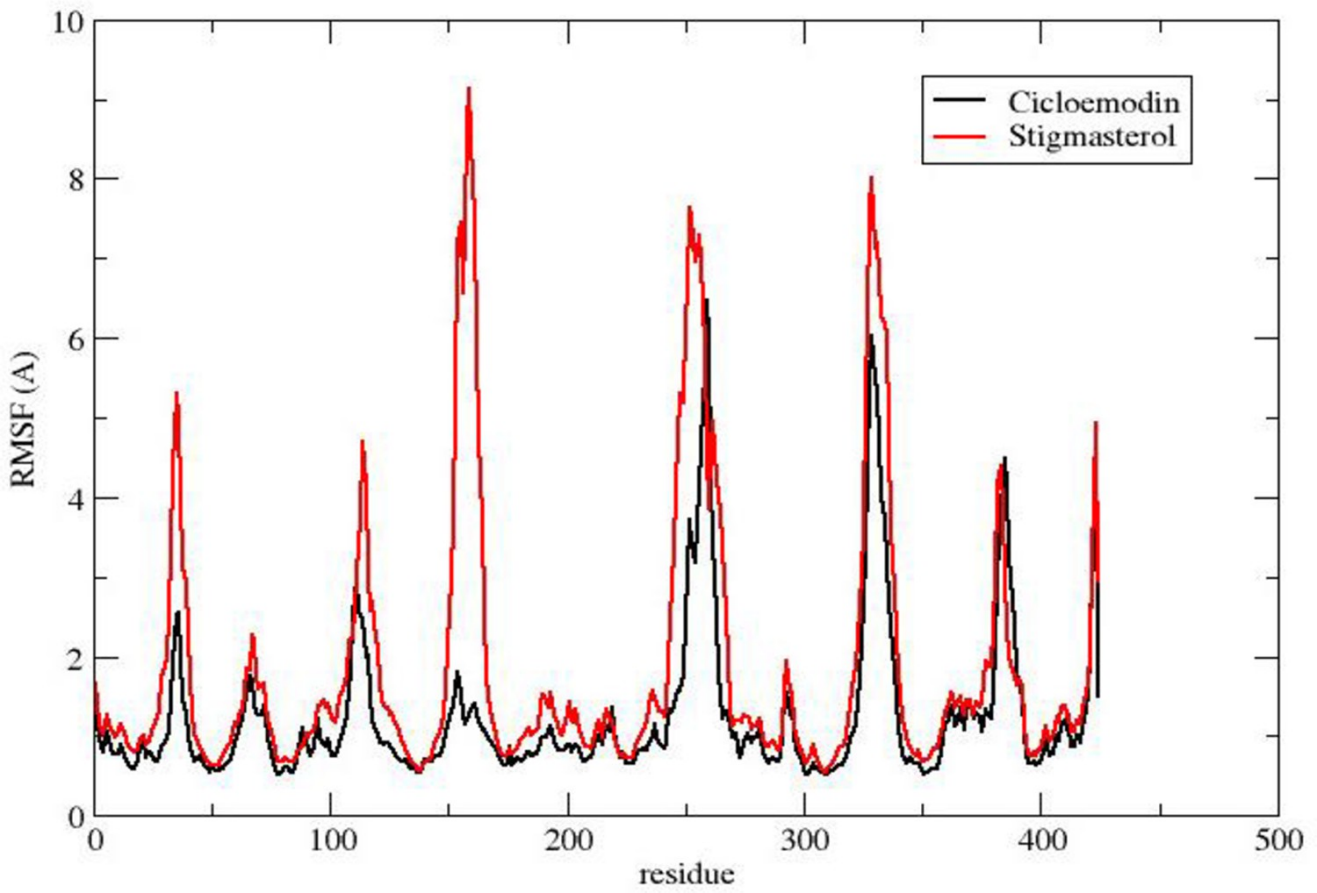
RMSF of active phytoconstituents in complexation with 1R9W.

The RMSF value reflects the particle’s deviation from the initial position of macromolecules in a protein’s three-dimensional structure. The RMSF plots of stigmasterol and clicoemodin in complexation with 1R9W revealed that the interactive amino acids of stigmasterol and clicoemodin have lesser fluctuations in the alpha-helix and beta-strand of the target protein (Fig 4).

**Fig 5:**
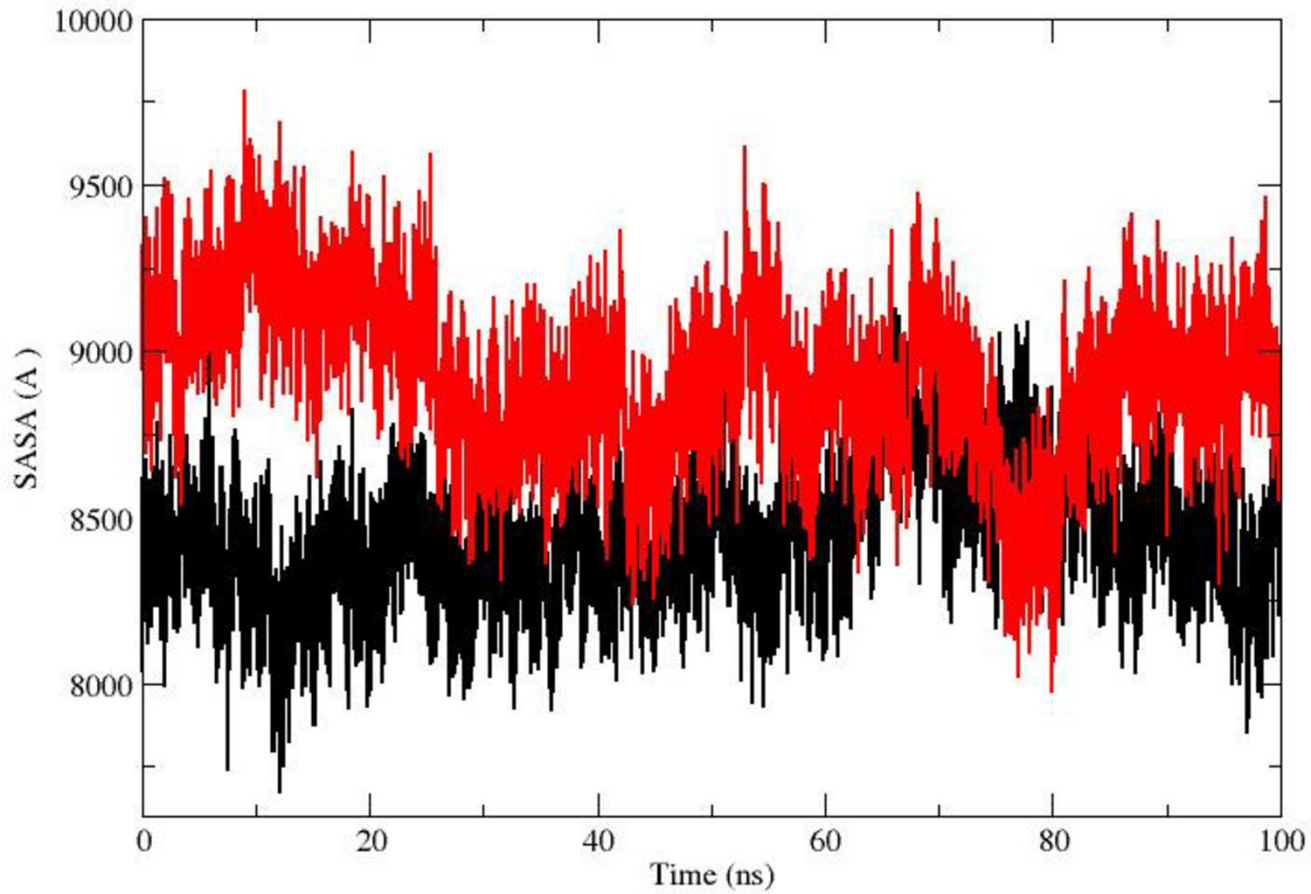
SASA of active phytoconstituents in complex with 1R9W: Black color is indicating clicoemodin and red color indicates stigmasterol.

**Fig 6:**
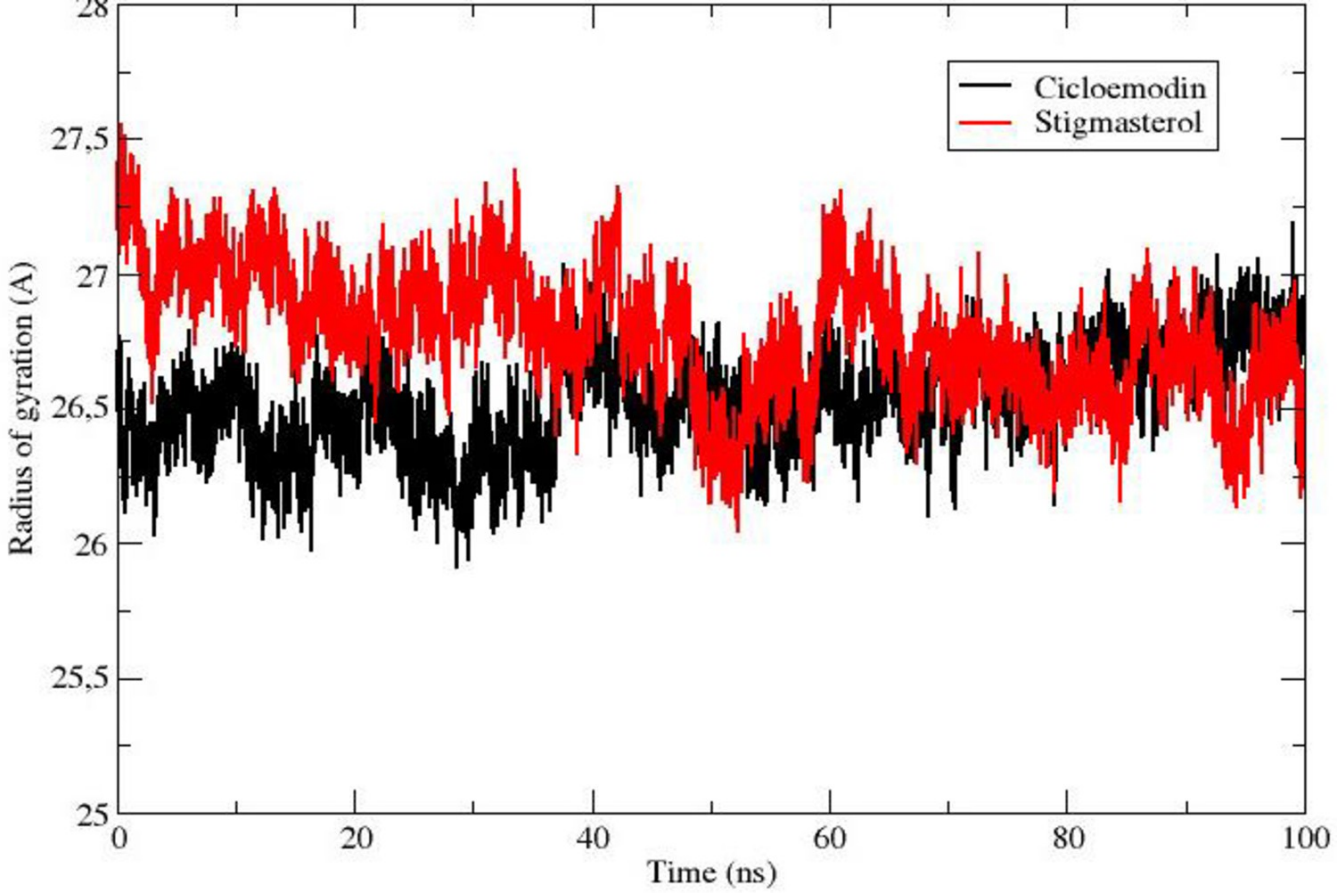
Radius of gyration of active phytoconstituents in complex with 1R9W: Black color is indicating clicoemodin and red color indicates stigmasterol.

The solvent-accessible range of stigmasterol is 8500–9500 Å, whereas in the case of clicoemodin, it lies between 8000–8500 Å (Fig 5). The radius of gyration plot established the compactness of stigmasterol and clicoemodin complexes with 1R9W, which confirms the stability of the protein-ligand complexes (Fig 6).

To understand the impact of inhibitors upon complexation in terms of their binding affinities, MM-PBSA binding free energy method was utilized to calculate the binding free energies and the energy components of their complexes are shown inTable 4.

**Table 4:** Binding free energies and their components for the two complexes: Clicoemodin-1R9W and stigmasterol-1R9W using the MM-PBSA method. The energy components are in kcal/mol.

Complex	Clicoemodin -2R5K	Stigmasterol -2R5K
Δ*E*_*vdW*_	− 45.61	− 33.09
Δ*E*_*el*_	− 39.38	− 10.88
Δ*G*_*PB*_	63.38	29.51
Δ*G*_*NP*_	-4.02	-3.67
Δ*G*_*bind*_	− 25.63	− 18.13

As it is evident in Table 4, the total binding free energies (ΔGbind) of clicoemodin-1R9W and stigmasterol-1R9W were -25.63 kcal/mol and -18.13 kcal/mol, respectively. Accordingly, among the two studied complexes, Clicoemodin-1R9W depicted the most favorable of ΔGbind with its lowest values of -25.63 kcal/mol. At this point, it is interesting to address the key contributions of each binding component that can impose to the total binding free energies.

## 4. Discussion

HPV has been described as the principal cause for the development of cervical cancer, and this potential is linked with its ability to produce various oncoproteins. Women infected by the human immunodeficiency virus (HIV) also have a very high susceptibility to HPV infections with the presence of multiple HPV types being typically reported [61]. HPV infections cause several benign proliferations including warts, epithelial cysts, anogenital, oro-laryngeal and -pharyngeal papillomas, intraepithelial neoplasias, keratoacanthomas, and other forms of hyperkeratoses. Brown and co-workers detected a high-risk HPV viral DNA on condylomata acuminata lesions from HIV immunosuppressed and otherwise healthy patients, always in association with a low-risk HPV type, HPV 6 or 11 [62].

In the last decades, the pharmaceutical industry has been employing computer-aided drug design (CADD) techniques to accelerate drug development, intending to reduce time, costs, and failures, mainly in the final stage [63, 64]. CADD attempts to improve the efficacy of the hit discovery process through in silico methodologies by testing and screening a large number of compounds to identify a small group of candidates with desirable pharmacological properties. The virus particle binds to its specific cell surface receptors, which allows it to enter the host cell. Virions can first bind to the basement membrane before being transferred to the basal keratinocyte cell surface [65, 66]. The structure-based approaches, including docking and the application of molecular dynamics simulations, use the 3D structure of the target molecule to screen potential ligands. These methods evaluate ligand recognition by the target molecule and the prediction and characterization of binding sites as well as their binding affinities [63, 67].

Computational-based approaches hold a great promise for improving drug development and revolutionizing clinical research by providing appropriate interventions for women diagnosed with cervical cancer. In the current study, we found that among all the selected one hundred nineteen phytoconstituents of the twenty unexplored medicinal plants of northwestern Himalaya, stigmasterol from Berberis aristata, and clicoemodin from Rheum emodi can be used as a potent drug to cure the HPV. In our previous research, Kumar et al. [68] studied the interactions of traditionally used phytoconstituents (ferulic acid, ursolic acid, jaceosidin, curcumin, gingerol, indol-3-carbinol, silymarin, resveratrol, berberine, and artemisinin) against E6 oncoprotein of high-risk HPV 18 by using Autodock 4.2 tool. Sookmai et al. [69] reported the anti-papillomavirus activity of *Clinacanthus nutans* extract; they also suggested C. nutans as prevention of HPV. Earlier a study of Moghadamtousi and co-workers, also showed a high potential of curcumin to cure high-risk HPV 16 and 18 [70]. Similarly, various researchers revealed the anticancerous potential of stigmasterol in the treatment of skin carcinoma [71], ovarian cancer [72], and gastric cancer [73, 74]. Previous reports also revealed that crude extracts or phytoconstituents obtained from medicinal plants of the Northwest Himalayas have various therapeutic attributes and show synergism of the in silico and in vitro investigations [75–83] and even Rolta et al. [84] reported the antiviral activity of phytoconstituents of important medicinal plants of the Northwest Himalayas against SARS-CoV-2. According to Cha et al. [85], emodin a major phytocompound of *Rheum emodi* inhibits the cell growth of the bladder cancer cell line. *Thymus serpyllum* essential oil and its two phytoconstituents thymol and carvacrol have antitumor activity against P815 mastocytoma cell line [86], HeLa (cervical carcinoma), and HepG2 (hepatocellular carcinoma) cells as reported by Hussain and co-workers, 2013 [87].

*In silico* docking examination by Muthukala et al. [88], has reported the inhibitory potential of quercetin and quercetin-like components towards human cervical cancer cell line proteins. Scheffner and coworkers also investigated that HPV oncoproteins i.e. E7 and E6 are activated in cervical cells during the progression of cervical cancer and also have been recognized to interact with tumor suppressor proteins viz. p53 and pRB respectively [89]. Through an *in-silico* approach, Kotadiya and Georrge [90] detected some putative drugs derived from the natural products against HPV, while Samant et al. [91] investigated the molecular interactions of HIV and anti-viral drugs targeting HPV-18 E6 protein. Inhibitory potential of natural products including colchicine, daphnoretin, curcumin, ellipticine, epigallocatechin-3-gallate, etc. towards HPV-16 E6 protein was also recognized by molecular docking [92]. Phytocompounds of *Ficus carica* is also reported as anticacinogenic drug [93]. Drug delivery systems can be developed to circumvent possible high toxicity and low availability of the compounds and, for instance, to combine this approach of L1 inhibitors with gene therapy to induce cancer cell apoptosis. Considering the costs of screening and treatment of cervical cancer and knowing that the highest incidence occurs in developing countries, a more cost-effective treatment is needed.

## 5. Conclusion

Major phytoconstituents of twenty traditionally important plants were screened against the HPV by molecular docking. Among the selected phytoconstituents stigmasterol from *Berberis aristata*, and clicoemodin from *Rheum emodi* were found to be the best inhibitors of HPV-18 (1R9W protein). ADME/T validated the proposal of stigmasterol and clicoemodin are safer drugs for the treatment of cervical cancer. MD simulations data showed that stigmasterol and clicoemodin are stable inside the binding pocket of the target protein. Since this study has only contemplated computational analysis, it is still necessary to validate the bioactivities of these important phytoconstituents *in vitro and in vivo* models before proposing their antiviral potential. We, also suggest that further research should take these results into account in both the discovery as well as the lead optimization stages to achieve the successful development of anti-HPV drugs.

## Supporting information

S1 Table Binding energy of selected phytocompounds with standard drugs. (DOCX)

S1 Fig Summary gastrointestinal (GI) absorption and blood-brain barrier (BBB) penetration, lipophilicity and polarity by using SwissADME platform. 1) 3-O-b-galactopyranoside, 2) 8-Oxyberberine, 3) 9-epoxylignan, 4) Apigenin, 5) Armatamide, 6) Berbamine, 7) Berberine, 8) Cannabigerol, 9) Cannabinol, 10) Catechin, 11) Chrysophanol, 12) Clicoemodin.
(TIF)

S2 Fig Summary gastrointestinal (GI) absorption and blood-brain barrier (BBB) penetration, lipophilicity and polarity by using SwissADME platform. 1) CurcubitacineE, 2) Emodin, 3) Eriodictyol-7-glucuronide, 4) Eriodictyol, 5) Glucomoringin, 6) Isovitexin, 7) Lupeol, 8) Oleanolic acid, 9) Pikuroside, 10) Piperolein B, 11) Pterygospermin, 12) Rubiadin.
(TIF)

S3 Fig Summary gastrointestinal (GI) absorption and blood-brain barrier (BBB) penetration, lipophilicity and polarity by using SwissADME platform. 1) Rutin, 2) Stigmasterol, 3) Taxifolin, 4) Tetrahydrocannabinol, 5) Thalicarpine, 6) Thalidasine, 7) Thalirugidine, 8) Thalirugine, 9) Thalisopine, 10) Thalrugosaminine B, 11) Podofilox, 12) Imiquimod.
(PNG)
