# Supplementary material for: Phytoconstituents of traditional Himalayan Herbs as potential inhibitors of Human Papillomavirus (HPV-18) for cervical cancer treatment: An In silico Approach
Source: PLoS One. 2022 Mar 17;17(3):e0265420. doi: 10.1371/journal.pone.0265420 (PMC8929605; doi:10.1371/journal.pone.0265420)
Supplement: S1 Table — (DOCX) [file pone.0265420.s001.docx]

**Table S1: Binding Energy of selected phytocompounds with standard drugs**

| **Phytocompounds/ Drugs** | **Binding energy (-kJ/mol)** |
| --- | --- |
| 3-O-b-galactopyranoside | -7 |
| 3-Octanone | -4 |
| 4-terpinol. | -5.2 |
| 8-Oxyberberine | -7.4 |
| 8-Prenylnaringenin | -6.7 |
| 9-epoxylignan | -7 |
| Alizarin | -6.8 |
| Aloe-emodin | -6.8 |
| Anthraquinone | -6.4 |
| Anthrarufin | -6.8 |
| Anthrone.pdbqt | -6.4 |
| Apigenin-7-O-glucoside | -8.2 |
| Apigenin-7-O-glucuronide | -8.1 |
| Apigenin | -7.1 |
| Armatamide | -7.2 |
| artemisinin | -6.6 |
| 𝛽-caryophyllene | -5.6 |
| Berbamine | -8.3 |
| Berberine | -6.9 |
| Bergapten | -5.6 |
| Bergenin | -6.4 |
| 𝛽-Bisabolene | -5.9 |
| Bornyl acetate | -5.3 |
| 𝛿-Cadinene | -5.8 |
| Camphene | -5.8 |
| Camphor | -5 |
| Cannabigerol | -7.2 |
| Cannabinoids | -5.9 |
| Cannabinol | -7.1 |
| Carvacrol | -5.4 |
| Caryophyllene oxide | -5.7 |
| 𝛽-Caryophyllene | -5.6 |
| Catechin | -7.5 |
| Chryophanol | -7 |
| Citral alpha | -4.5 |
| citronellal | -4.7 |
| Clicoemodin | -8.4 |
| Columbamine | -6.6 |
| Curcubitacine E | -7.7 |
| Dactylorhin E (1) | -6.7 |
| Dantron | -6.7 |
| Dihydroartemisinic acid | -5.8 |
| Elemicin | -4.8 |
| Emodin | -7.1 |
| Eriodictyol-7-glucuronide | -9.1 |
| Eriodictyol | -7.5 |
| Gallic acid | -5.4 |
| Geraniol | -4.9 |
| Geranyl acetate | -4.5 |
| Glucomoringin | -7.4 |
| Guaiol | -6.4 |
| Isocorydine | -6.4 |
| Isoelemicin | -4.8 |
| Isovitexin | -7.9 |
| Jatrorrhizine | -6.7 |
| Juglone | -5.5 |
| Lambertine | -6.6 |
| lemonene | -4.9 |
| Linalool | -4.7 |
| Lupeol | -8.2 |
| Meso-dihydroguaiaretic acid | -5.9 |
| methoxyeugenol | -4.8 |
| Methyl a-L-arabinopyranoside | -4.5 |
| Myrcene | -4.3 |
| Myristicin | -5 |
| Nerol | -4.3 |
| Niazinicin A | -5.8 |
| Niazinin | -6.1 |
| Noroxyhydrastinine | -5.7 |
| Oleanolic acid | -8 |
| Oleic acid | -4.5 |
| Oxyberberine | -6.9 |
| Oxycanthine | -8 |
| Palmatine | -6.5 |
| Palmitic acid | -4.2 |
| Physicon | -6.6 |
| Piceatannol | -6.5 |
| Pikuroside | -7.7 |
| 𝛽-Pinene | -5.1 |
| 𝛼-Pinene | -5.7 |
| Piperazine | -3.1 |
| Piperine. | -6.8 |
| Piperolein A | -6.8 |
| Piperolein B | -6.9 |
| Podophyllin | -5.4 |
| Pterygospermin | -7.6 |
| Pyrogallol | -4.7 |
| Quinizarin | -6.6 |
| Rubiadin | -6.9 |
| Rutin | -8.3 |
| Sabinene | -5 |
| Safrole | -5 |
| Sedanolide | -5.5 |
| Sesamin | -7.5 |
| Spathulenol | -6.5 |
| Stigmasterol | -8.7 |
| Tambulin | -7 |
| Taxifolin | -7.2 |
| terpinolene | -5 |
| Tetrahydrocannabinol | -7.3 |
| Thalicarpine | -7.9 |
| Thalidasine | -7.8 |
| Thalirugidine | -8.4 |
| Thalirugine | -8 |
| Thalisopine | -8.1 |
| Thalrugosaminine | -7.8 |
| Thalrugosidine | -8.3 |
| Thymol | -5.3 |
| Trans-Nerolidol | -5.2 |
| Trimyristin | -4.9 |
| Verbascoside | -7.8 |
| Xanthyletin | -6.7 |
| β-Sitosterol-β-D-Glucoside | -7.7 |
| Podofilok | -6.9 |
| Imiquimod] | -6.1 |
