# Supplementary figures and images for: Phytoconstituents of traditional Himalayan Herbs as potential inhibitors of Human Papillomavirus (HPV-18) for cervical cancer treatment: An In silico Approach
Source: PLoS One. 2022 Mar 17;17(3):e0265420. doi: 10.1371/journal.pone.0265420 (PMC8929605; doi:10.1371/journal.pone.0265420)

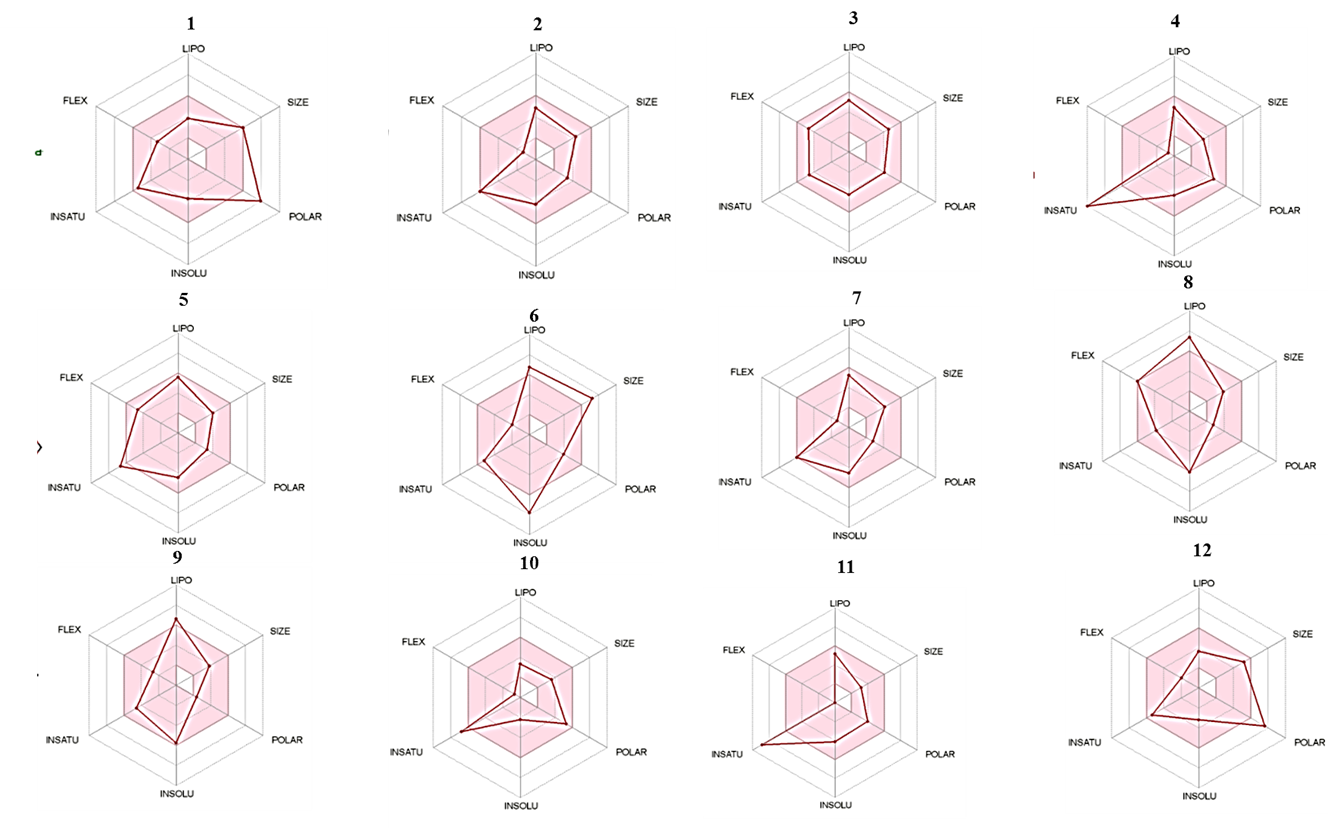

Supplement: S1 Fig — 1) 3-O-b-galactopyranoside, 2) 8-Oxyberberine, 3) 9-epoxylignan, 4) Apigenin, 5) Armatamide, 6) Berbamine, 7) Berberine, 8) Cannabigerol, 9) Cannabinol, 10) Catechin, 11) Chrysophanol, 12) Clicoemodin. (TIF) [file pone.0265420.s002.tif]

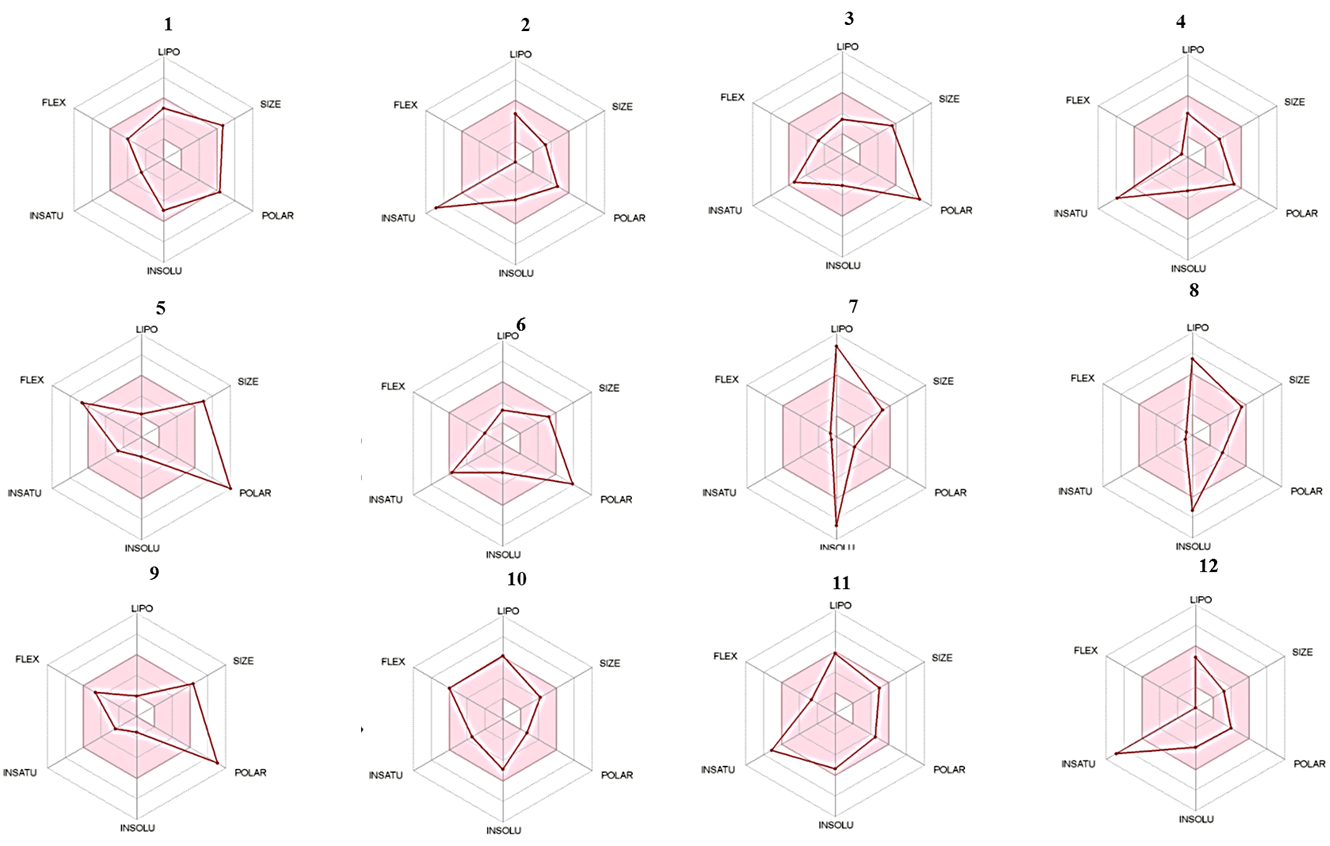

Supplement: S2 Fig — 1) CurcubitacineE, 2) Emodin, 3) Eriodictyol-7-glucuronide, 4) Eriodictyol, 5) Glucomoringin, 6) Isovitexin, 7) Lupeol, 8) Oleanolic acid, 9) Pikuroside, 10) Piperolein B, 11) Pterygospermin, 12) Rubiadin. (TIF) [file pone.0265420.s003.tif]

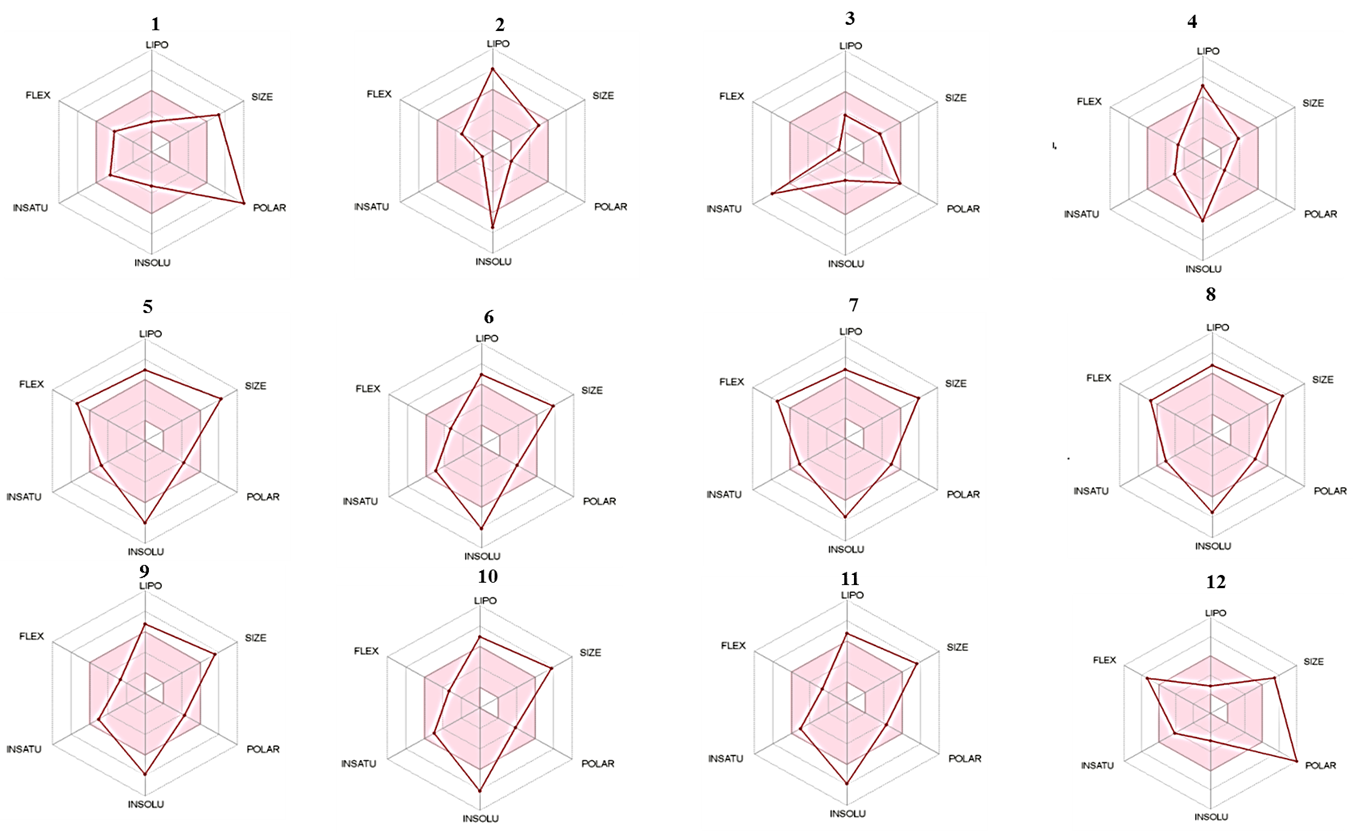

Supplement: S3 Fig — 1) Rutin, 2) Stigmasterol, 3) Taxifolin, 4) Tetrahydrocannabinol, 5) Thalicarpine, 6) Thalidasine, 7) Thalirugidine, 8) Thalirugine, 9) Thalisopine, 10) Thalrugosaminine B, 11) Podofilox, 12) Imiquimod. (PNG) [file pone.0265420.s004.png]
